# Phonetic relevance and phonemic grouping of speech in the automatic detection of Parkinson’s Disease

**DOI:** 10.1038/s41598-019-55271-y

**Published:** 2019-12-13

**Authors:** Laureano Moro-Velazquez, Jorge A. Gomez-Garcia, Juan I. Godino-Llorente, Francisco Grandas-Perez, Stefanie Shattuck-Hufnagel, Virginia Yagüe-Jimenez, Najim Dehak

**Affiliations:** 1 Johns Hopkins University, Department of Electrical and Computer Engineering, Baltimore, 21218 USA; 2 Universidad Politécnica de Madrid, Escuela Técnica Superior de Ingeniería y Sistemas de Telecomunicación, Madrid, 28031 Spain; 3 Hospital General Universitario Gregorio Marãnón, Madrid, 28007 Spain; 4 Massachusetts Institute of Technology, Speech Communication Group, Cambridge, 02139 USA; 5 Consejo Superior de Investigaciones Científicas, Centro de Tecnologías Físicas Leonardo Torres Quevedo, Madrid, 28006 Spain

**Keywords:** Diagnostic markers, Parkinson's disease, Parkinson's disease

## Abstract

Literature documents the impact of Parkinson’s Disease (PD) on speech but no study has analyzed in detail the importance of the distinct phonemic groups for the automatic identification of the disease. This study presents new approaches that are evaluated in three different corpora containing speakers suffering from PD with two main objectives: to investigate the influence of the different phonemic groups in the detection of PD and to propose more accurate detection schemes employing speech. The proposed methodology uses GMM-UBM classifiers combined with a technique introduced in this paper called phonemic grouping, that permits observation of the differences in accuracy depending on the manner of articulation. Cross-validation results reach accuracies between 85% and 94% with AUC ranging from 0.91 to 0.98, while cross-corpora trials yield accuracies between 75% and 82% with AUC between 0.84 and 0.95, depending on the corpus. This is the first work analyzing the generalization properties of the proposed approaches employing cross-corpora trials and reaching high accuracies. Among the different phonemic groups, results suggest that plosives, vowels and fricatives are the most relevant acoustic segments for the detection of PD with the proposed schemes. In addition, the use of text-dependent utterances leads to more consistent and accurate models.

## Introduction

Parkinson’s Disease (PD) is a chronic condition caused by the gradual death of brain cells, including those located in the *substantia nigra*, implicated in the production of dopamine. This neurotransmitter is involved in many neuronal activities that play a determining role in motor tasks. The consequent loss of dopamine in the patient affected by PD results in a lack of coordination, muscle rigidity and slowness of movements, among other signs.

The most common criteria for PD diagnosis are mainly based on the observation of motor *cardinal signs*^1^, non-motor indicators such as dementia, depression, excessive salivation and constipation and other physiological and cognitive manifestations whose evaluation is employed in clinical diagnosis. Notwithstanding, neuropathological diagnosis during autopsy is considered the gold standard, although some studies demonstrate that following the usual clinical diagnosis criteria it is possible to obtain 90% accuracy in a final judgment within an average time of 2.9 years^2^.

Recent studies point toward the development of new neuro-protective therapies that will potentially slow or stop the progression of the disease^3^. When these therapies are ready, new tools to support and reduce diagnosis time or even provide an early detection of the disorder are going to be crucial. Additionally, reducing the time to diagnosis might improve and maintain the patient’s quality of life and increase their life expectancy^4^. The search for these new tools can be considered highly relevant since, unfortunately, there are currently no efficient, reliable methods capable of achieving an early or fast diagnosis in most of the cases, due to the fact that the symptoms of PD often overlap with symptoms of other diseases.

Introducing new objective methods for automatic assessment employing the speech signal can help reduce the diagnosis time^5^, and speech is particularly useful for these purposes because it requires very precise and complex movements. These movements are usually affected early by the neurodegenerative processes associated with PD, resulting in dysphonia, dysarthria and disprosody^6–9^. For instance, several studies have reported lower amplitude and velocity in jaw and lower lip opening during articulation of PD patients in comparison to controls^10–14^. Furthermore, PD affects different phonemic groups in distinct ways, with stop-plosives, fricatives and affricates the most affected, as some early works performing a perceptual analysis of parkinsonian speech suggest^15–19^. However these studies have not determined whether there are differences between the phonemic groups for patients vs. controls which are not easily perceptible to human listeners, but which can nevertheless be relevant for early detection.

Some preliminary findings suggest that this might be the case. For example, it has been found that voiced segments tend to be longer, while stop silences produced by the closures before bursts tend to disappear, in the speech of PD patients compared to control speakers^7,20^. These observations are in direct relationship with misarticulation phenomena common in some dysarthrias by which plosives are produced as fricatives, where the frication noise is not necessarily preceded by a closure. This phoneme transformation phenomenon is known as spirantization. On the other hand, differences in the slopes and variability of the formant frequencies between patients and controls (and especially between the vowel space areas (VSA) of both groups) have been reported^21–27^. Based on that, recent studies propose automatic systems to detect or assess PD making use of the articulatory aspects of speech and advanced signal processing techniques, suggesting that speech processing can derive powerful indicators of imprecise consonant articulation in PD-related dysarthria^20,21,28–30^. The accuracy in PD detection of these works, as in most of the works in the literature, does not exceed 90%, although it is difficult to compare the performance of the different methodologies since each study uses a different corpus and evaluates its results following a different procedure. However, although some studies^31^ analyze the importance of several words or segments in respect to others in PD detection, none of the works found in the literature have studied in detail the detection capabilities as a function of the manner classes of phonemes, that is to say, their manner of articulation. This may be crucial for determining the focus of future systems and to adequately select the speech tasks to be employed.

**Figure 1:**
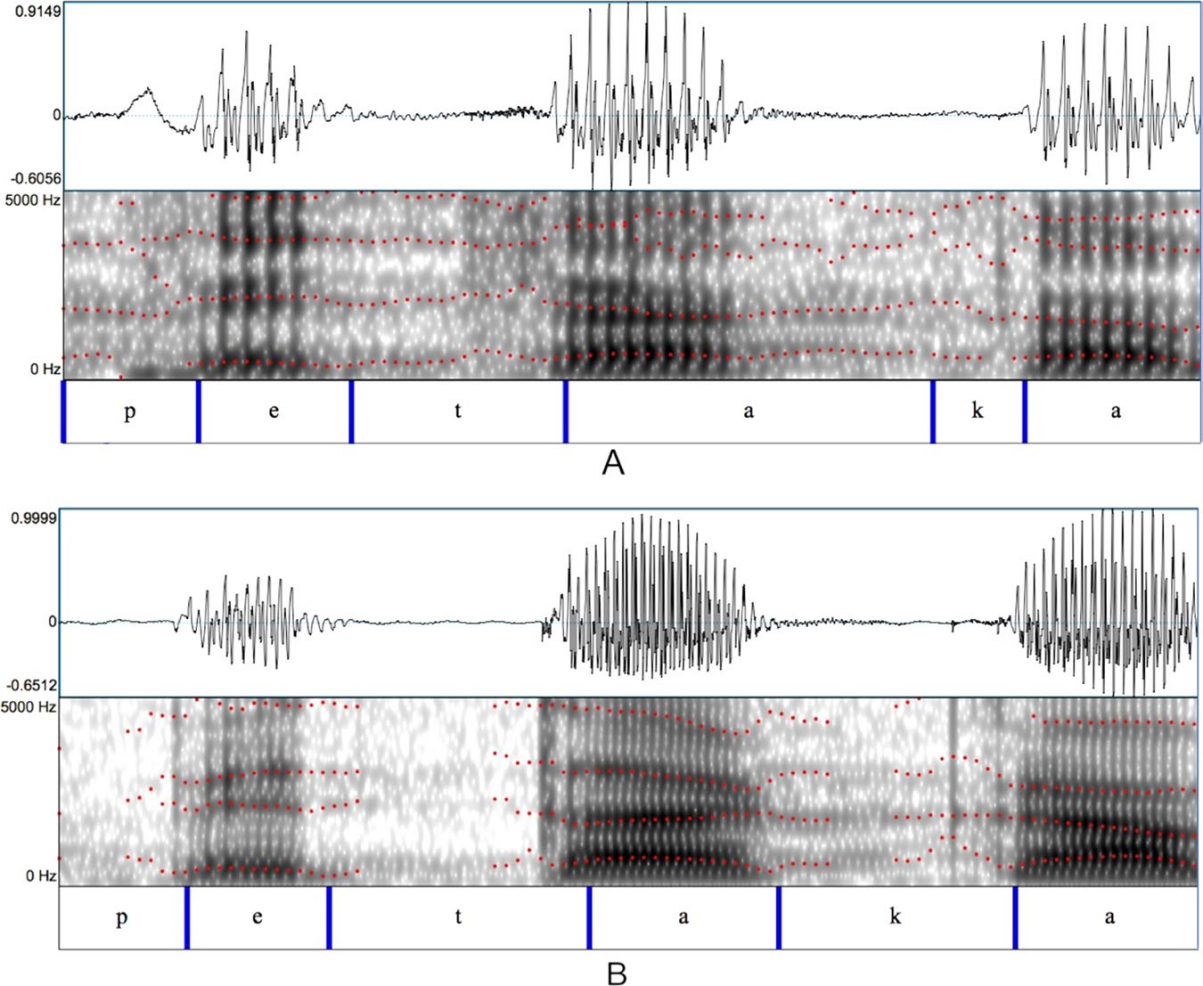
Waveforms and spectrograms of a speaker with PD (newly diagnosed) and a control speaker pronouncing the word [petaka]. Obtained from the Neurovoz corpus^39^. Red dot-lines mark the first four formants calculated with Praat software^46^. (**A**) Idiopathic PD female speaker. Age: 59. UPDRS: 9. Span: 720 ms. (**B**) Control female speaker. Age: 59. Span: 857 ms

**Figure 2:**
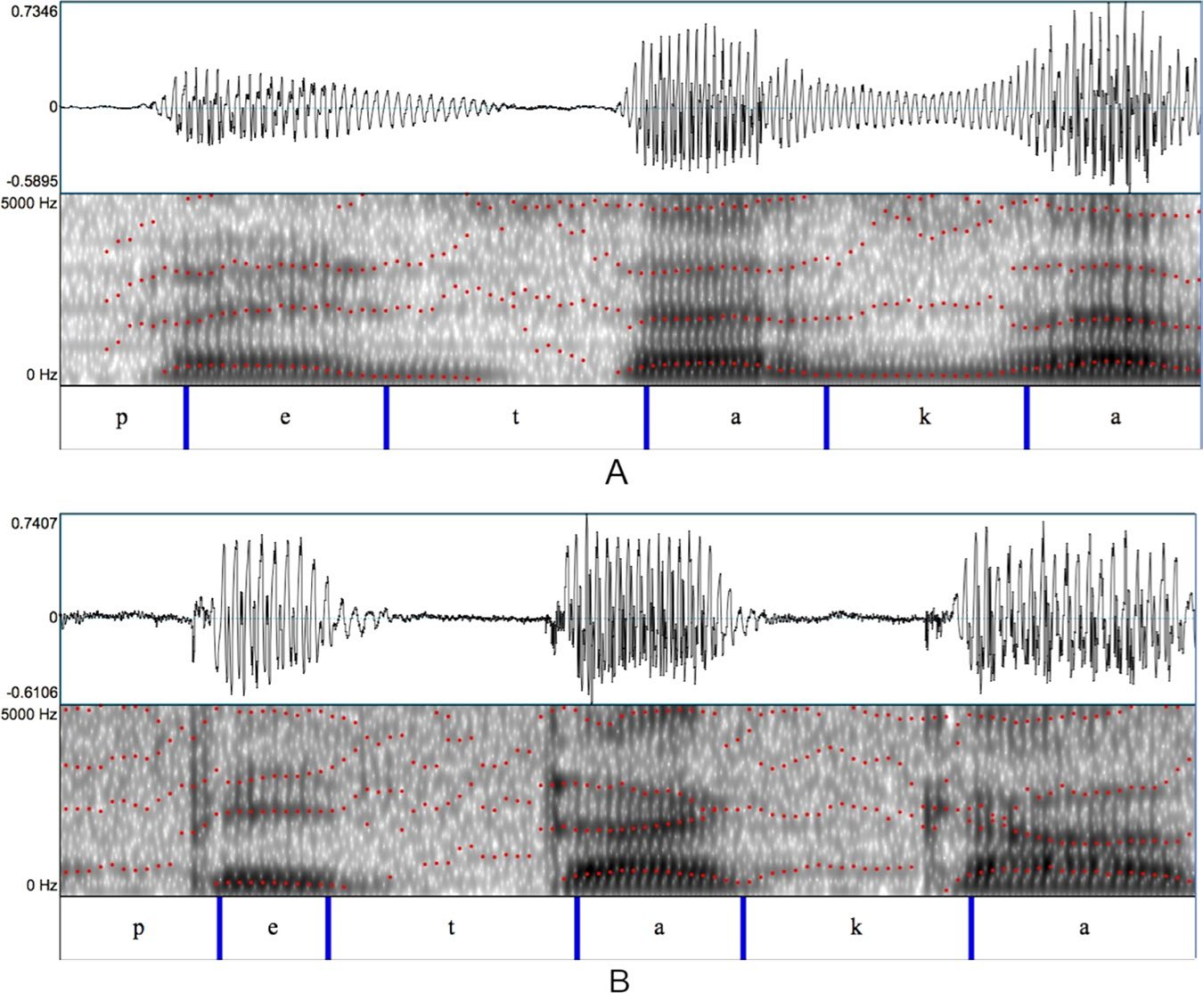
Waveforms and spectrograms of a speaker with PD (in an advanced stage) and a control speaker pronouncing the word [petaka]. Obtained from the Neurovoz corpus. Red dot-lines mark the first four formants calculated with Praat software. (**A**) Idiopathic PD female speaker. Age: 85. UPDRS: 47. Span: 810 ms. (**B**) Control female speaker. Age: 83. Span: 780 ms.

In this respect, Figs. 1 and 2 allow a comparison between the waveforms and spectrograms of two parkinsonian and two control speakers while pronouncing the word “petaca” ([petaka], *flask* in English) containing three plosives and extracted from a longer sentence. In Fig. 1, a newly diagnosed PD patient with a low Unified Parkinson’s Disease Rating Scale (UPDRS)^32^ motor examination score (part III) is compared with an age-matched control (the rating according to the UPDRS motor examination, whose values can range between 0 and 72, is accomplished through clinical observations of the patient’s movements.) Although both waveforms exhibit a silence or stop closure between the end of the vowels and the beginning of the plosives, the spectrogram shows a tendency for the patient to convert the release bursts of plosives into a more gradual articulation. This effect is not observed in the control speaker, whose bursts are clearly visible and delimited in the spectrogram. More pronounced effects can be found in Fig. 2, where the stop silences are shorter in the patient (before [t]) or nonexistent (before [k]), transforming the plosive consonant [k] into something similar to a fricative [G]. The periodic signal substituting for the stop silence before the consonant burst reveals an incomplete lip closure or a possible lack of control of the glottal source which keeps vibrating when it should have stopped. These effects are visible in the patient’s spectrogram too, where the bursts of the plosives are almost indistinguishable and, in the case of the plosive [k], the first spectral peak from the preceding vowel [a] is joined with the first spectral peak from the following vowel [a].

In cases such as these, a separate statistical modeling of the acoustic characteristics of plosive segments from patients and controls would lead to substantially different probabilistic densities. Hence, this work proposes different approaches to automatically detect PD while extending the analysis to different types of acoustic segments for this task.

## Overview and Contribution

This work presents a method for studying the importance of the different phonemic groups in the automatic detection of PD through the analysis of the speech signal, in order to expand our knowledge of how PD affects speech. Therefore, one of the goals of this study is to identify the speech segments that are more relevant in automatic detection systems, serving as well to determine more appropriate speech tasks to be employed in this detection. We hypothesize that not all types of acoustic segments have the same relevance in the detection, since each one derives from a different narrowing, articulation and configuration of the vocal tract.

Although the literature shows some examples of phonetic and phonemic analysis of parkinsonian speech, a thorough study of the relevance of the different types of phonemic segments (defined here as different manner classes) in the automatic detection of PD has not yet been carried out. To analyze this relevance and to confirm the influence of PD in the different manners of articulation, several PD detection approaches were analyzed in the present study, making use of Gaussian Mixture Model-Universal Background Model (GMM-UBM) techniques^33^ and Perceptual Linear Predictive (PLP) features^34^ as described in a previous study^35^ but using only certain phonemic categories from the speech signal. These acoustic segments were selected depending on the manner of articulation, after applying the *phonemic grouping* process presented in this study to the speech signal. For these purposes, state-of-the-art speech forced alignment techniques were used.

In addition, the approaches described in this work were tested on three different parkinsonian speech corpora, in order to determine their generalization properties.

## Theoretical Background

### Phonetic and phonemic considerations

The term ‘phoneme’ refers to abstract units which distinguish one word from another in a language, while the different pronunciation variants of a phoneme are often referred to as allophones. Different categorizations of allophones of the Spanish language can be found in the literature, from which the ones proposed by Quilis^36^ are widely used. The present study uses his categorization of manner of articulation, since this is related to the type of articulatory movements and the degree of narrowing of the vocal tract during the production of each allophone.

Focusing on this categorization, it is possible to find two main types of segments: vowels and consonants, where consonants can be divided into plosives, fricatives, affricates, liquids and nasals (also known as *manner classes*). The plosive consonants are those preceded by a stop or a total obstruction of the articulators that results in pressure buildup behind the constriction, producing a burst of noise after its release. An example of a plosive is the [′t] in typical productions of the word “pastel” [pas’tel]. Fricatives are those sounds in which the constriction is incomplete, so that air passing through the narrow but incomplete constrictions generates turbulence noise, and there is no previous stop closure. An example of fricative is the [′f] in the word “alféizar” [al’fejθar]. Affricates are those sounds which begin with a stop closure, have a noise burst which is extended, via a lengthened incomplete constriction, as frication. An example is the [′t∫] in the word “colchón” [kol’t∫on]. Liquids are similar to fricatives in the sense that they involve a narrowing in the vocal tract, but in this case the articulators do not approach closely enough to produce the same turbulence noise as in fricatives. The [′ř] in the word “rey” [′řei] is an example of a liquid. Nasal consonants are produced when there is a constriction in the oral tract, and the soft palate is lowered to allow the air coming from the larynx to pass through the nasal cavities and escape through the nose. In Spanish, nasals are sonorants, which means that the glottal source is functioning while articulating, as in vowels. An example of a nasal is the [′n] in the word “canario” [kanaɾjo].

### Forced alignment and phonemic grouping

Speech forced alignment techniques^37^ are used to identify and label sound within a speech recording when its transcription is known. This process consists in the automatic segmentation of the signal, giving as a result separated speech acoustic segments. These segments are often referred to as allophones or context-appropriate pronunciation variants of the phonemes that specify the word forms (although the acoustic segments that are identified can also be described as interlandmark intervals, because their boundaries are often determined by abrupt changes in the acoustic signal known as landmarks^38^.) Forced alignment methods produce a segmentation of the signal, with each identified interval labeled as a single allophone determined by the transcription, no matter how they were realized in the surface phonetics of the signal. Figures 1 and 2 show an example of a forced alignment of the word “petaca”.

The speech forced alignment set-up described by Moro *et al*.^39^ was employed in this work to train a Forced Alignment Model (FAM). This FAM can be used to perform the phonetic segmentation and labeling of the speech recordings. Then, this labeling can be employed to identify the speech segments that correspond to a certain manner category, that is to say, to group together only acoustic segments that correspond to either affricates, fricatives, liquids, nasals, plosives or vowels. This process, consisting of the automatic selection of groups of acoustic segments that share a manner of articulation has been called *phonemic grouping*. It permits the analysis of the acoustic differences between speakers with and without PD regarding different types of vocal tract constrictions (plosives, fricatives and liquids), the vibration of the vocal folds in combination with articulatory movements (vowels, liquids and nasals) and the articulation of the soft palate (nasals). This helped to test if the poor motor control of patients with Parkinson’s disease would result in more variability for all manner classes.

## Materials and Methodology

### Materials: speech corpora

Five speech corpora were used in this study: Neurovoz, GITA, CzechPD, FisherSP and Albayzin. The first three are made up of different speech tasks from PD patients and matched control speakers. Albayzin is an auxiliary corpus used to train the different UBM as explained in Methods subsection while FisherSP was employed to create a FAM^39^.

#### Neurovoz

This corpus contains 47 parkinsonian and 32 control speakers whose mother tongue is Spanish Castillian. The sub-set utilized in the present study contains a Diadochokinetic (DDK) task (repetitions of the sylable sequence “pa-ta-ka”), six text-dependent utterances (TDU) and a monologue (picture description). The speech was produced at a comfortable phonatory level. Table 1 contains the transcription and International Phonetic Alphabet (IPA) transcription of the TDU. All of the patients were under pharmacological treatment and took the medication between 2 and 5 h before the speech recording^39^. The Ethics Committee of Hospital General Universitario Gregorio Marañón approved the recording of the speech and the associated experimental protocols and methods, according to the Helsinki Declaration developed by the World Medical Association and derived European Directives. Signed informed consent was obtained from all speakers.

**Table 1:** Spanish transcription of the six Neurovoz TDU (Spanish), IPA transcription and translation to English.

Sentence #	Spanish transcription/IPA transcription/*English translation*
1	Cuando las barbas de tu vecino veas pelar, pon las tuyas a remojar/**[kwa**$\mathop{{\bf{n}}}\limits_{\cap }$**do la**$\mathop{{\bf{s}}}\limits_{\cup }$ **βaɾβa**$\mathop{{\bf{s}}}\limits_{\cup }$ $\mathop{{\boldsymbol{\eth}}}\limits_{\top }$**e tu βeθino βea**$\mathop{{\bf{s}}}\limits_{\cup }$ **pelaɾ pon la**$\mathop{{\bf{s}}}\limits_{\cup }$ **tuʝa**$\mathop{{\bf{s}}}\limits_{\cup }$ **a řemoxaɾ]**/*When your neighbor’s beard you see peeling, put yours to soak*
2	De la calle vendrá quien de tu casa te echará/**[de la kaʝe Be**$\mathop{{\bf{n}}}\limits_{\sqcap }$**dɾa kjen** $\mathop{{\boldsymbol{\eth}}}\limits_{\top }$**e tu kasa te e**$\mathop{{\bf{t}}{\boldsymbol{\int }}}\limits^{\frown {}}$**aɾa]**/*From outside will come that who will kick you out from your house*
3	Cuando el diablo no sabe qué hacer, con el rabo mata moscas/**[kwa**$\mathop{{\bf{n}}}\limits_{\cap }$**do el** $\mathop{{\boldsymbol{\eth}}}\limits_{\top }$**jaβlo no saβe ke aθeɾ kon el řabo mata moskas]**/*When the devil does not know what to do, it kills flies with its tail*
4	La petaca blanca es mía/**[la petaka βlan**^**ɣ**^**ka e**$\mathop{{\bf{s}}}\limits_{\cup }$ **mia]**/*The white flask is mine*
5	No pidas a quien pidió, ni sirvas a quien sirvió/**no pi**$\mathop{{\boldsymbol{\eth}}}\limits_{\top }$**a**$\mathop{{\bf{s}}}\limits_{\cup }$ **a kjen pi**$\mathop{{\boldsymbol{\eth}}}\limits_{\top }$**jo ni siɾβa**$\mathop{{\bf{s}}}\limits_{\cup }$ **a kjen siɾβjo]**/*Do not beg the one who begged, nor serve the person who served*
6	El que a buen árbol se arrima, buena sombra le cobija/**[el ke a βwen aɾβol se ařima βwena sombɾa le koβixa]**/*To the one that comes to a good tree, good shade covers him*

#### Gita

This corpus contains a variety of speech tasks from 50 PD patients and 50 control speakers whose native language is Spanish Colombian^40^. Three types of speech tasks from GITA were utilized in this study: a DDK task (“pa-ta-ka”), six TDU and a monologue. Table 2 contains the transcription and International Phonetic Alphabet (IPA) transcription of the TDU. The recording of this corpus and the associated experiments are in compliance with the Helsinki Declaration and were approved by the Ethics Committee of the Clínica Noel, in Medellín, Colombia. A written informed consent was signed by each participant according to the authors of the corpus^40^.

**Table 2:** Transcription of the six GITA TDU (Spanish), IPA transcription and translation to English.

Sentence #	Spanish transcription/IPA transcription/*English translation*
1	Luisa Rey compra el colchón duro que tanto le gusta/**[lwisa ře i kompɾa el kol’tʃon** $\mathop{{\boldsymbol{\eth}}}\limits_{\top}$**uɾo ke tanto le ɣusta]**/*Luisa Rey buys the hard mattress that she so much likes*
2	Los libros nuevos no caben en la mesa de la oficina/**[loʂ li**$\mathop{{\boldsymbol{\beta }}}\limits_{\top }$**ɾoʂ ’nwe**$\mathop{{\boldsymbol{\beta }}}\limits_{\top }$**oʂ no ka**$\mathop{{\boldsymbol{\beta }}}\limits_{\top }$**en en la meʂa** $\mathop{{\boldsymbol{\eth}}}\limits_{\top }$**e la ofi’ʂina]**/*The new books do not fit in the office desk*
3	Laura sube al tren que pasa/**[la**$\mathop{{\bf{u}}}\limits_{\cap }$**ɾa suβe al tɾen ke pasa]**/*Laura gets on the passing train*
4	Mi casa tiene tres cuartos/**[mi kasa tjene tɾe**$\mathop{{\bf{s}}}\limits_{\cup }$ **kwartos]**/*My house has three rooms*
5	Omar, que vive cerca, trajo miel/**[õmar ke βiβe seɾka traxo mjel]**/*Omar, living nearby, brought honey*
6	Rosita Niño, que pinta bien, donó sus cuadros ayer/**[řosita niɲo ke pi**$\mathop{{\bf{n}}}\limits_{\cap }$**ta βjen** $\mathop{{\boldsymbol{\eth}}}\limits_{\top }$**ono sus kwa**$\mathop{{\boldsymbol{\eth}}}\limits_{\top }$**ɾos aʝeɾ]**/*Rosita Niño, who paints well, donated her paintings yesterday*

#### CzechPD

The CzechPD subset employed in this study only contains a DDK task (repetitions of the syllable sequence “pa-ta-ka”) from 20 newly diagnosed and untreated speakers with PD and 14 controls whose mother tongue is Czech^21^. This subset only contains male speakers. The recording of this corpus and associated experiments are in compliance with the Helsinki Declaration and were approved by the Ethics Committee of the General University Hospital in Prague. All participants provided written informed consent, according to the authors of the corpus^28^.

Table 3 shows the age, sex, UPDRS and years since diagnosis statistics of speakers in the three corpora.

**Table 3:** Demographic statistics of Neurovoz, GITA and CzechPD corpora.

	Neurovoz				GITA				CzechPD	
	Female		Male		Female		Male		Male	
	PD	Ctrl	PD	Ctrl	PD	Ctrl	PD	Ctrl	PD	Ctrl
#Subjects	18	18	29	14	25	25	25	25	20	16
Age, average	70.9 (8.4)	68.4 (6.0)	71.9 (12.3)	66.6 (6.4)	60.7 (7.3)	61.4 (7.0)	61.5 (11.6)	60.5 (11.6)	61 (11.7)	61.8 (12.9)
Age range	59–86	58–83	41–88	55–77	49–75	49–76	33–81	31–86	34–83	36–80
UPDRS ^*^, average	16.9 (11.5)	—	19.6 (11.8)	—	37.5 (14.0)	—	37.7 (22.0)	—	17.9 (7.1)	—
Years since diagnosis	6.4 (6.4)	—	7.6 (4.7)	—	12.6 (11.5)	—	8.9 (5.9)	—	2.4 (1.6)	—

Ages are expressed in years. Ctrl stands for healthy controls. Standard deviation values are presented in parenthesis. *The Neurovoz corpus only contains UPDRS part III, i. e. motor examination; GITA contains global values of Movement Disorder Society UPDRS; CzechPD contains global values of UPDRS.

#### Auxiliary corpora

The *phonetic dataset* from the Albayzin corpus^41^ is also employed in the present study. This phonetically balanced dataset, sampled at 16 kHz and quantized with 16 bits, contains more than 4.800 utterances (4.1 h) in Castillian Spanish along with their transcriptions.

In addition, the FisherSP (Fisher Spanish) corpus, recorded by the Linguistic Data Consortium (8 kHz as sampling rate and 16 bits) to train and evaluate automatic speech recognizers in the Spanish language, was used in this study. It comprises around 163 h of telephonic speech from native Spanish speakers from more than 20 countries, along with their transcriptions.

### Methodology

The general methodology of this study followed these main steps:

- Firstly, some trials employing different speaker recognition technologies were performed following the procedure analyzed in a previous study^35^ in order to set a **baseline** to be compared with the proposed new approaches.
- Then, a **FAM in the Spanish language** was trained with FisherSP and used to segment and label all the utterances with associated transcriptions from three corpora: GITA, Neurovoz and Albayzin.
- The labeling was employed to identify tokens of the different manner classes in these three corpora, and to create several GMM-UBM models, employing Albayzin as UBM and the parkinsonian corpora for adaptation. **Three different approaches** employing phonemic grouping are proposed to analyze the importance of the different phoneme categories for the automatic detection of PD. When possible, some trials using CzechPD to adapt the UBM were also carried out.
- Finally, several **cross-corpora trials** employing the baseline procedure and some of the proposed approaches completed and validated the proposed methodology.

#### General considerations

In all the proposed approaches, the same front-end was used; utterances were filtered and downsampled to 16 kHz if their sampling frequency was higher. Then, the signals were normalized and characterized using Rasta-PLP + *derivatives* (Δ + ΔΔ)^34^, with number of PLP coefficients (*F*) varying in the range {10 ... 20} in steps of 2. The length of the frames was set to 15 ms with an overlapping of 50%, employing a Hamming window and 5 coefficients in the FIR filter used to calculate derivatives as this is the set-up that led to optimum results in a previous study^35^. In the baseline and in the new proposed approaches, all the available TDU were pooled to train the same model for all of the trials associated to one specific corpus. Therefore, the studied allophones were not sentence-dependent. The cross-validation trials followed a k-folds strategy (11 folds). None of the utterances or frames from a speaker employed to adapt the UBM were used in the testing stage during the cross-validation.

Lastly, and regarding the classification stage employing GMM-UBM in the baseline and the proposed approaches, the number of Gaussians *G* varied in powers of 2 from 4 to 256.

#### Baseline

In the baseline trials, all the available speech tasks from each parkinsonian corpus were used to adapt several UBM trained with Albayzin. An UBM is a GMM model that estimates the probability density function that characterizes a group of feature vectors of dimension *D* from a certain corpus, using a linear combination of *G* Gaussian components. This UBM serves as an initialization of the final GMM-UBM model that is used, in this case, to detect PD. In this work the resulting GMM-UBM were obtained through Maximum a Posteriori (MAP) adaptation of the UBM employing each parkinsonian corpus separately, a method that is similar to some speaker recognition systems^33^. This methodology provided accuracies up to 85% in the cited previous study^35^.

#### Proposed approaches and phonemic grouping

Several types of trials were carried out, employing only the specific acoustic segments in the speech signal that correspond to a single intended manner class (identified by means of phonemic grouping), to train the UBM and to adapt it following MAP adaptation. Three different approaches were followed, depending on where the phonemic grouping process was applied: in the adaptation-testing set (GITA, Neurovoz or CzechPD), in the UBM corpus or in both. On each trial, the GMM-UBM were first adapted and then tested using only one specific parkinsonian corpus and acoustic segments associated with only one manner class: either fricative, liquid, nasal, plosive or vowel. Affricate segments were not analyzed, as these are underrepresented in TDU from GITA and Neurovoz (see Table 4). One of the main reasons this group is unrepresented is that among all of the manner classes discussed here, affricates are the least common class in the Spanish language, representing less than 3% of the total phonemes^42^.

**Table 4:** Total number of repetitions of consonants and vowels in TDU from GITA and Neurovoz.

#Repetitions		
Phonemic category	GITA	Neurovoz
Affricate	2	3
Fricative	29	35
Liquid	23	26
Nasal	19	21
Plosive	26	35
Vowels	85	107

In order to categorize the acoustic segments phonemically into groups that each correspond to a single intended manner class, a Spanish FAM^39^ was created in Kaldi^43^ and then used to segment and label GITA, Neurovoz and Albayzin. Then, after calculating the feature vectors containing *D* Rasta-PLP + Δ + ΔΔ coefficients for all the frames of the speech utterances, these were distributed into the corresponding manner groupings according to their phonemic labels, for training, adaptation or testing, in the corpora in which phonemic grouping was applied, depending on the experiment.

**Figure 3:**
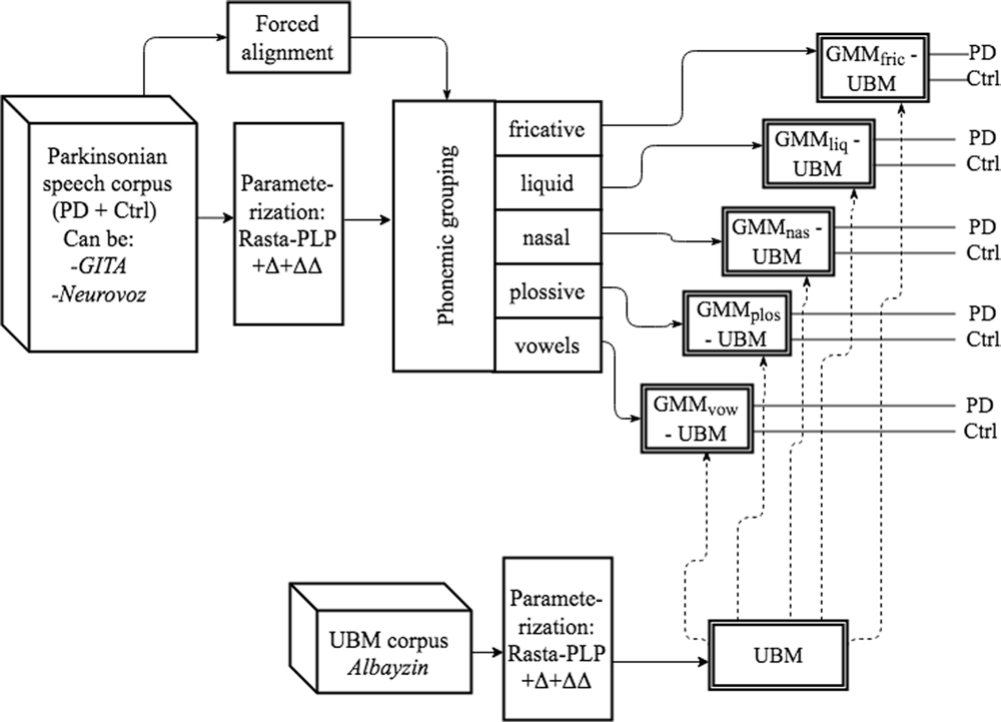
First proposed approach^5^. Phonemic grouping methodology is applied to the parkinsonian corpus (*raw-phon*).

In the first approach (*raw-phon*), the phonemic grouping was applied only to the TDU of the adaptation-testing corpora (Neurovoz and GITA, used separately). A depiction of this approach is shown in Fig. 3. The notation *raw-phon* indicates that the we did not apply the phonemic grouping process to the UBM corpus (*raw*) while we applied it to the adaptation corpus (*phon*).

**Figure 4:**
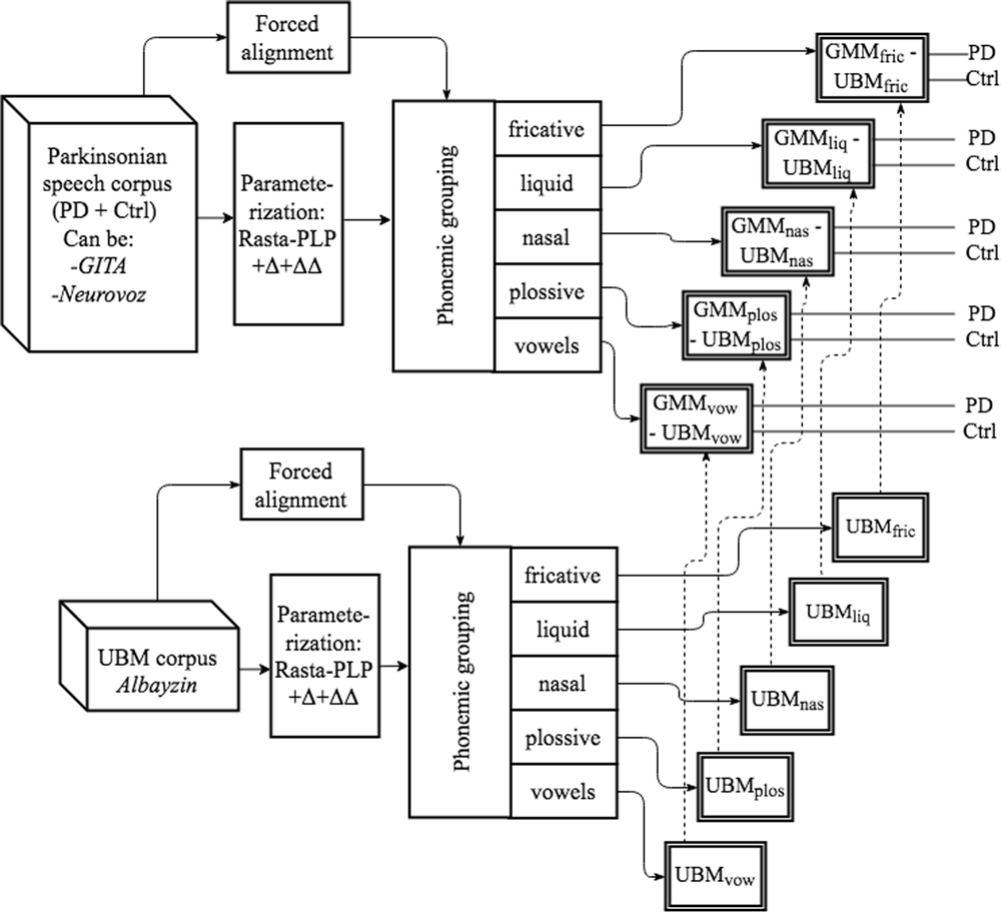
Second proposed approach^5^. Phonemic grouping methodology is applied to the parkinsonian and UBM corpora (*phon-phon*).

In the second approach (*phon-phon*), a new round of trials was carried out following the same premises but pursuing now phonemic grouping in the UBM too (Albayzin). A depiction of the second approach is presented in Fig. 4.

It is convenient to remark that in these two first approaches the phonemic grouping was applied to both training (adaptation) and testing utterances in the parkinsonian corpora. In this way, the obtained systems modeled only one phonemic manner category at a time (fricative, plosive, etc.) and during the testing stage, only the specific acoustic segments associated with each category were employed. Therefore, the two first approaches and their associated rounds of trials permitted analysis of the importance of the different phonemic manner categories in the automatic detection of PD using connected speech. In these two first approaches, only TDU from GITA and Neurovoz were employed, since these were the only recordings that included transcriptions.

**Figure 5:**
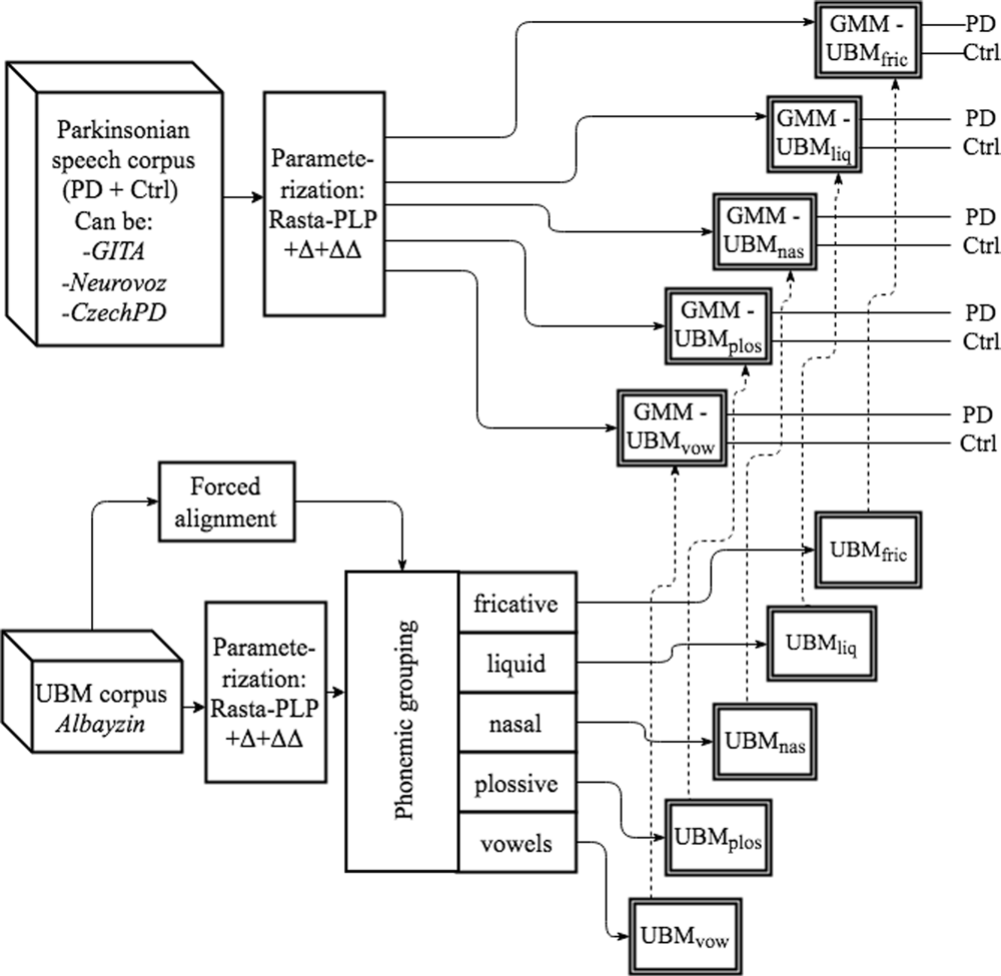
Third proposed approach^5^. Phonemic grouping methodology is applied to the UBM corpus (*phon-raw*).

In the third approach (*phon-raw*), the phonemic grouping was applied only to the UBM corpus in order to analyze the importance of the initialization of the GMM-UBM. In this last approach, all three parkinsonian corpora were employed without performing any forced alignment. TDU, monologues, and DDK tasks from GITA and Neurovoz were examined separately. The DDK task from CzechPD was employed too since it was considered that this task has similar phonetic characteristics in the three parkinsonian corpora and can be used to adapt the UBM, independently of the mother tongue of the speaker (Spanish or Czech). Figure 5 shows a diagram of the third approach.

**Figure 6:**
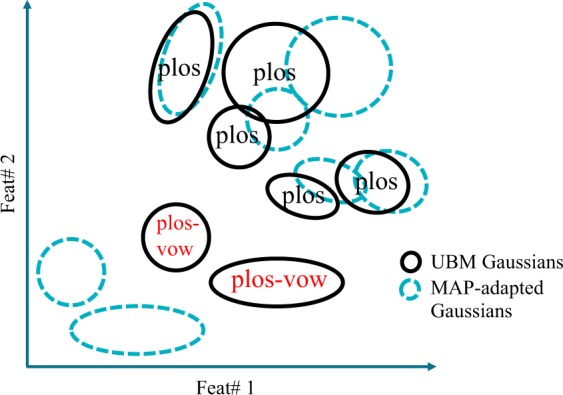
Representation of Gaussians in the third approach (*phon-raw*). The GMM-UBM in the example contains 7 Gaussians modelling 2 features. Plosive grouping was applied to the UBM which was adapted with all the frames from a DDK task.

The objective of this last approach was to provide GMM-UBM classifiers that are more precise in modeling a certain type of acoustic segment but without completely discarding the rest of the acoustic segments present in the adaptation subset; in this way the last approach was different from the two previous proposed approaches. First, we consider that after a plosive grouping (as for any other phonemic grouping) of the UBM corpus we will obtain two types of acoustic segments: those including only frames related to plosives and those containing frames that also have information about adjacent sounds. This last type of frames arises near the initial or ending parts of a plosive, where the frame may include part of the beginning (or ending) of the plosive and part of the adjacent sound, when the frame coincides with a transition into or out of an adjacent vowel. Considering this, Fig. 6 illustrates an example of plosive grouping only in the UBM. In this example, most of the UBM Gaussians in the upper part of the figure -five in this case- have been modeled using only plosives (plos) whereas the other two UBM Gaussians in the lower part arose from the less abundant frames that contained information about the plosives along with information about other adjacent acoustic segments -mainly vowels- (plos-vow). Considering the use of a DDK task (“pa-ta-ka”) as speech material for the MAP adaptation, our hypothesis is that the sufficient statistics^33^ obtained from the plosive segments present in the adaptation utterances ([p], [t], [k]) tend to perform the adaptation of the Gaussians created in the UBM with only plosives. On the other hand, the sufficient statistics obtained from the remaining segments ([a]) tend to adapt the other two Gaussians from the UBM, that are closer to those segments. Thus, the resulting GMM-UBM is modelling the features coming from several types of acoustic segments but is more focused on the plosives. Consequently, and generalizing, the phonemic grouping of the UBM corpus produces GMM-UBM models oriented to either fricatives, liquids, nasals, plosives or vowels, depending on the phonemic grouping but considering also the rest of the acoustic segments to a minor extent.

#### Scoring

The score for each utterance *u* comprising *N* frames with respect to the GMM-UBM relative to class *c* (**Γ**^*c*^) was calculated employing the log-likelihood of the feature vectors from every frame **x**_*n*_ as:

1 $${\Lambda }_{u}^{c}=\frac{1}{N}\mathop{\sum }\limits_{n\mathrm{=1}}^{N}\,\log \,p({{\bf{x}}}_{n}|{{\boldsymbol{\Gamma }}}^{c})\,,$$

where *p*(**x**_*n*_|**Γ**^*c*^) is the Gaussian density of class *c* (*c* can be PD or Ctrl) for feature vector **x**_*n*_.

Finally, the global scores for each utterance were expressed in the form of log-likelihood ratio:

2 $${\Lambda }_{u}={\Lambda }_{u}^{{\rm{PD}}}-{\Lambda }_{u}^{{\rm{Ctrl}}}.$$

To compute the class membership of a certain utterance from the test set, its score was compared with a threshold, *λ* to prove the hypothesis of this utterance belonging to the parkinsonian class, *H*_PD_. If the score Λ_*u*_ was higher than *λ*, the hypothesis was accepted; otherwise, the hypothesis was rejected. In all the approaches analyzed in this study, this threshold was determined by the equal error rate (EER)^44^ point obtained with the scores of the adaptation data.

#### Fusion of scores

After analyzing the results of all the approaches, a fusion of scores from the approach providing the best accuracy and Area Under the ROC Curve (AUC) was studied. For each corpus, the speaker scores obtained from the five possible phonemic groupings (fricative, liquid, nasal, plosive and vowels) were fused following all the possible combinations of *n*−*tuples*, going from 2−*tuples* to 5−*tuples*. To obtain a final score coming from the fusion of several scores, a logistic regression was employed. Therefore, for a given speaker and speech task a new score was calculated considering between two and five scores from this speaker and task, each score coming from a different phonemic grouping. Given that *F* is the number of PLP coefficients and *G* is the number of Gaussians on the GMM, only the scores produced with the same *F* and *G* were combined. For instance, to obtain the fricative-liquid-vowels score fusion for a certain trial, the three single scores from each speaker for the fricative, liquid and vowel categories, respectively, obtained with the same *F* and *G* were used in the fusion.

#### Cross-corpora validation

**Figure 7:**
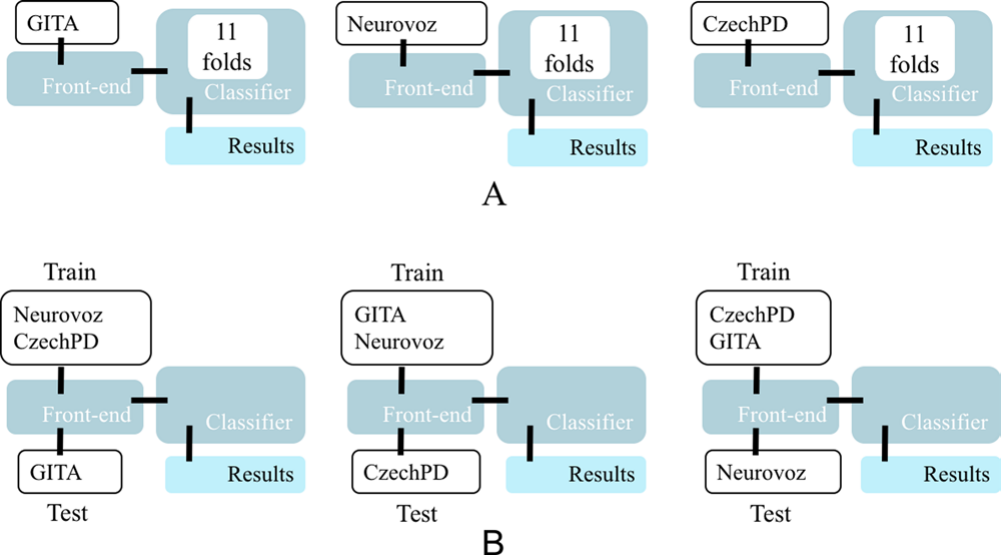
Diagram of trials. In the diagram, the classifiers are GMM-UBM where the UBM is trained with Albayzin. (**A**) Scheme of cross-validation trials (11 folds). The classifiers can be referred to any type of phonemic grouping (fricative, liquid, nasal, plosive or vowels) or approach (baseline, *raw-phon, phon-phon* or *phon-raw*). (**B**) Scheme of cross-corpora trials. The classifiers can be referred to any type of phonemic grouping. Only the baseline and the proposed approach leading to the best results in cross-validation trials were used in cross-corpora trials.

Finally, a cross-corpora validation procedure was followed considering the baseline and the third approach (*phon-raw*), in which we applied the phonemic grouping process to the Albayzin corpus to obtain the five different types of UBM, that were subsequently adapted and tested with the DDK tasks from the parkinsonian corpora. In particular, three rounds of trials were carried out: in each one, two of the corpora were used jointly to adapt the model and the remaining corpus was utilized exclusively for testing. Therefore, there were several models created with the speakers from GITA and Neurovoz and tested with CzechPD; other models created with GITA and CzechPD and tested with Neurovoz; and a third group of models adapted employing the utterances from Neurovoz and CzechPD and tested with GITA. In this case, the same front-end, classification parameters and scoring procedures of the rest of the experimental set were used. The differences between cross-validation and cross-corpora validations is illustrated in Fig. 7.

## Results

In this section, the results of the cross-validation (k-folds) and cross-corpora trials are expressed in terms of accuracy (%) ± Confidence Interval (CI)^44^, AUC, sensitivity and specificity. To calculate the CI, 95% confidence level was considered. In all tables, best global results per corpus are in bold.

Table 5 contains the results of baseline trials (in which no phonemic grouping was applied to any utterance) employing the three parkinsonian corpora separately. Table 6 includes the best results of the three proposed approaches with GITA and Neurovoz and considering different speech tasks. In the first approach (*raw-phon*), phonemic grouping was applied only to the parkinsonian corpora; in the second (*phon-phon*) the phonemic grouping was applied to the parkinsonian and to the UBM corpus (Albayzin); and in the third approach (*phon-raw*), the phonemic grouping was applied only to the UBM corpus. Additionally, Table 7 includes the best results employing CzechPD in the third approach. In this case, only the DDK task was used.

**Table 5:** Results for the **baseline** scheme with no phonemic grouping.

Speech task	GITA		Neurovoz		CzechPD	
	Accu. ± CI	AUC	Accu ± CI	AUC	Accu. ± CI	AUC
**TDU**	79 ± 8	0.86	86 ± 8	0.93	—	—
**DDK**	81 ± 8	0.88	79 ± 9	0.85	88 ± 12	0.94
**Monol**.	80 ± 8	0.88	72 ± 13	0.79	—	—

**Table 6:** Results for the **three approaches** employing **GITA** and **Neurovoz** to adapt the UBM created with Albayzin.

		GITA				Neurovoz			
Approach (speech task)	Phonemic group.	Accu. ± CI	AUC	Sens.	Spec.	Accu. ± CI	AUC	Sens.	Spec.
**1** *raw-phon* **(TDU)**	**Fricatives**	77 ± 8	0.85	0.72	0.82	83 ± 9	0.88	0.86	0.78
	**Liquids**	77 ± 8	0.84	0.77	0.78	82 ± 9	0.91	0.81	0.83
	**Nasals**	77 ± 8	0.81	0.7	0.84	82 ± 9	0.89	0.83	0.78
	**Plosives**	84 ± 7	0.9	0.83	0.84	83 ± 9	0.94	0.81	0.87
	**Vowels**	81 ± 8	0.89	0.74	0.88	77 ± 10	0.71	0.87	0.89
**2** *phon-phon* **(TDU)**	**Fricatives**	79 ± 8	0.86	0.74	0.84	86 ± 8	0.87	0.88	0.83
	**Liquids**	77 ± 8	0.83	0.79	0.76	83 ± 9	0.9	0.81	0.87
	**Nasals**	77 ± 8	0.84	0.79	0.76	82 ± 9	0.9	0.83	0.78
	**Plosives**	85 ± 7	0.89	0.81	0.88	85 ± 9	0.93	0.81	0.91
	**Vowels**	86 ± 7	0.9	0.79	0.92	77 ± 10	0.89	0.76	0.78
**3** *phon-raw* **(TDU)**	**Fricatives**	82 ± 8	0.89	0.82	0.82	**89** ± **7**	**0.93**	**0.87**	**0.91**
	**Liquids**	81 ± 8	0.88	0.74	0.88	87 ± 7	0.93	0.87	0.88
	**Nasals**	82 ± 8	0.88	0.82	0.82	85 ± 8	0.93	0.85	0.84
	**Plosives**	**85** ± **7**	**0.91**	**0.82**	**0.88**	86 ± 8	0.92	0.85	0.88
	**Vowels**	82 ± 8	0.89	0.76	0.88	85 ± 8	0.92	0.83	0.88
**3** *phon-raw* **(DDK)**	**Fricatives**	80 ± 8	0.87	0.8	0.8	83 ± 9	0.9	0.89	0.73
	**Liquids**	82 ± 8	0.87	0.8	0.84	81 ± 9	0.89	0.85	0.73
	**Nasals**	83 ± 7	0.89	0.86	0.8	82 ± 9	0.87	0.85	0.77
	**Plosives**	82 ± 8	0.88	0.86	0.78	86 ± 8	0.88	0.89	0.81
	**Vowels**	83 ± 7	0.88	0.86	0.8	81 ± 9	0.88	0.87	0.69
**3** *phon-raw* **(Monol.)**	**Fricatives**	80 ± 8	0.87	0.71	0.88	74 ± 12	0.77	0.47	0.9
	**Liquids**	80 ± 8	0.87	0.73	0.86	66 ± 14	0.77	0.06	1
	**Nasals**	77 ± 8	0.84	0.69	0.84	70 ± 13	0.65	0.41	0.87
	**Plosives**	80 ± 8	0.88	0.71	0.88	70 ± 13	0.73	0.41	0.87
	**Vowels**	78 ± 8	0.84	0.76	0.8	72 ± 13	0.74	0.65	0.77

**Table 7:** Results for the **third approach** employing **CzechPD** (with no phonemic grouping) to adapt the UBM created with Albayzin. Only the DDK task is considered.

Speech task	Phonemic group.	Accuracy ± CI	AUC	Sens.	Spec.
**DDK**	**Fricatives**	**94** ± **1**	**0.96**	**0.9**	**1**
	**Liquids**	94 ± 1	0.95	0.9	1
	**Nasals**	91 ± 1	0.95	0.9	0.93
	**Plosives**	91 ± 1	0.98	0.9	0.93
	**Vowels**	91 ± 1	0.96	0.9	0.93

**Figure 8:**
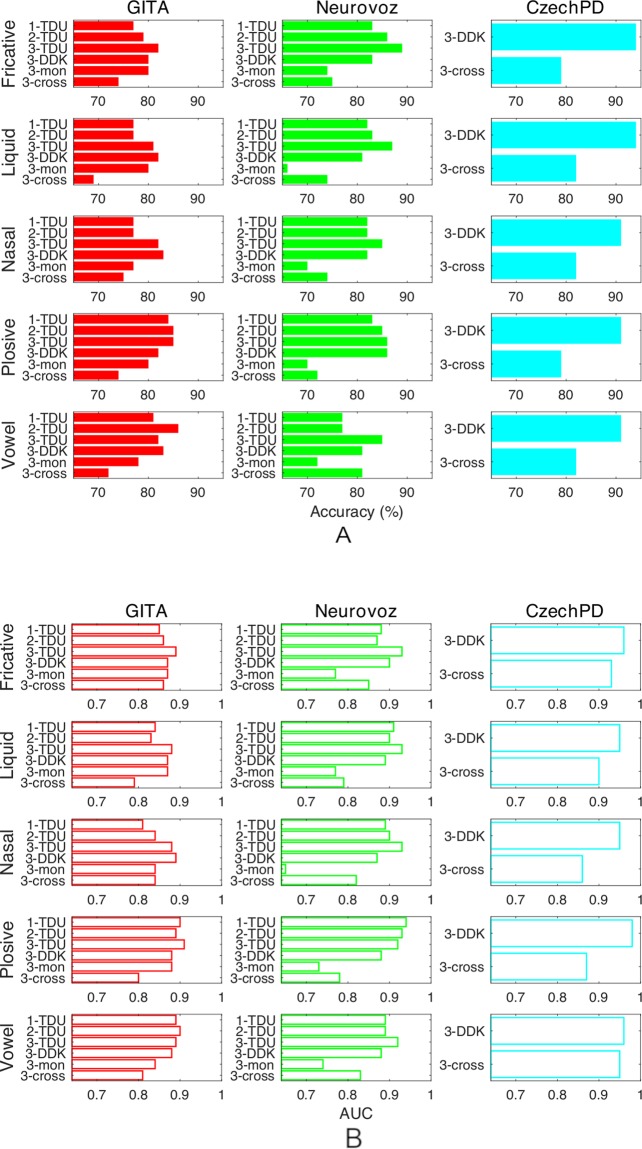
Best accuracies (**A**) and AUC (**B**). Results are referred to the three proposed approaches (marked as 1, 2 or 3) and speech tasks, where *mon* stands for monologues and *cross* for cross-corpora trials.

Table 8 shows the results of the fusion of scores of the different phonemic groupings in the third approach (*phon-raw*) since this is the one that leads to higher accuracies in the cross-validation trials, according to Table 6. Finally, Tables 9 and 10 show the results of cross-corpora trials in the baseline scheme and in the third approach, respectively. Figure 8 includes a graphical representation of best accuracies and AUC reached in the different trials to compare the relevance of each phonemic category in the automatic detection.

**Table 8:** Best results after the fusion of scores for the three parkinsonian corpora separately.The scores were obtained using the third approach (*phon-raw*).

Corpus	Speech task	Combination	Accu. ± CI	AUC
**GITA**	**TDU**	plosive-liquid	84 ± 7	0.9
	**DDK**	nasal-liquid	83 ± 7	0.88
	**Monol**.	liquid-vowel	82 ± 8	0.89
**Neurovoz**	**TDU**	fricative-vowel	**89** ± **7**	**0.95**
	**DDK**	liquid-vowel	83 ± 9	0.89
	**Monol**.	plosive-nasal-vowel	77 ± 12	0.79
**CzechPD**	**DDK**	fricative-nasal	**94** ± **6**	**0.98**

**Table 9:** Cross-corpora results in GITA, Neurovoz and CzechPD, employing Albayzin for the UBM (Baseline). For every trial, two parkinsonian corpora were used for training and the remaining, for testing.

Test corpus	Speech task	Accu. ± CI	AUC	Sens.	Spec.
**GITA**	**DDK**	73 ± 9	0.82	0.84	0.62
**Neurovoz**	**DDK**	75 ± 10	0.82	0.8	0.65
**CzechPD**	**DDK**	79 ± 14	0.91	1	0.5

**Table 10:** Cross-corpora results in GITA, Neurovoz and CzechPD, employing Albayzin for the UBM with the five different types of phonemic grouping.*Phon-raw* approach was employed. For every trial, two parkinsonian corpora were used for adaptation of the UBM and the remaining, for testing.

Test corpus	Phonemic group.	Accu. ± CI	AUC	Sens.	Spec.
**GITA**	**Fricatives**	74 ± 9	0.86	0.66	0.82
	**Liquids**	69 ± 9	0.79	0.94	0.44
	**Nasals**	75 ± 8	0.84	0.86	0.64
	**Plosives**	74 ± 9	0.8	0.86	0.62
	**Vowels**	72 ± 9	0.81	0.7	0.74
**Neurovoz**	**Fricatives**	75 ± 10	0.85	0.76	0.73
	**Liquids**	74 ± 10	0.79	0.87	0.5
	**Nasals**	74 ± 10	0.82	0.78	0.65
	**Plosives**	72 ± 10	0.78	0.93	0.35
	**Vowels**	81 ± 9	0.83	0.91	0.62
**CzechPD**	**Fricatives**	79 ± 14	0.93	0.9	0.64
	**Liquids**	82 ± 13	0.9	0.9	0.71
	**Nasals**	82 ± 13	0.86	0.95	0.64
	**Plosives**	79 ± 14	0.87	0.95	0.57
	**Vowels**	82 ± 13	0.95	0.85	0.79

## Discussion

In this study, three different approaches based on a GMM-UBM classification scheme were tested with two main objectives: to study the relevance of different phonemic groups in the automatic detection of PD and to provide new detection schemes. For each proposed approach, a phonemic grouping based on manner of articulation (fricative, liquid, nasal, plosive or vowel) was applied to the parkinsonian corpora or to the UBM corpus (Albayzin), enabling the observation of changes in accuracy and AUC depending on the employed phonemic manner category.

In general, the CI of the accuracy values generates overlapping margins in the results. This is a common issue in studies employing a small number of speakers in comparison to other works addressing speech or speaker recognition problems in which hundreds or even thousands of subjects are analyzed. The reason for this reduced number of speakers is related to the limited number of PD patients in a hospital, who are both willing to collaborate and meet the inclusion criteria, and to the cost of the resources needed to engage more patients from different institutions.

### Phonemic grouping

In the first two approaches (*raw-phon* and *phon-phon*), only TDU from GITA and Neurovoz are employed since these are the only tasks including transcription, which is needed for the forced alignment processes. Regarding these approaches, Table 6 and Fig. 8 show that best results are obtained for the plosive and vowel categories in GITA, and the fricative and plosive categories in Neurovoz. Therefore, these results point towards a higher relevance of the plosive segments of speech from parkinsonan patients in automatic detection using speech with the proposed methodologies.

Regarding the third approach (*phon-raw*), the speech tasks used are TDU, DDK and monologues from GITA and Neurovoz and DDK from CzechPD, since in this case no speech forced alignment is needed in the parkinsonian corpora as the phonemic grouping is applied only to the UBM corpus. When the adaptation-testing corpus is GITA in this approach, the best AUC and accuracy are obtained employing plosives, vowels and fricatives with TDU and monologues. Something different occurs in the Neurovoz corpus, where the fricative phonemic grouping in the UBM always produces the best AUC and accuracy, followed by the plosive and vowel categories. In this same corpus, nasal and vowel categories in the UBM corpus provide the best results when employing a DDK task, followed closely by plosives. With respect to CzechPD, fricatives provide the highest accuracy of the experimental set (94%), while plosives yield the highest AUC (0.98), as indicated in Table 7. Table 6 and Fig. 8 suggest that the plosive grouping has a similar behavior in GITA and Neurovoz and provides high accuracy, AUC and sensitivity in most of the approaches. The other phonemic groupings have unequal results. For instance, while the fricative grouping outperforms the other phonemic groupings in Neurovoz using the third approach, it does not provide these good results in GITA.

In general, although Czech and Neurovoz are class-unbalanced, the observed sensitivity and specificity rarely differ by more than 0.10 absolute points in the results shown in the cited result tables.

The fusion of scores using logistic regression produces moderate improvements in the trials with Neurovoz where the maximum AUC goes from 0.93 to 0.95 for TDU. In the remaining cases, fusion does not produce any increase of the accuracy or AUC. This suggests that there is not complementary information among the scores of the different systems. Regarding the cross-corpora trials in which the phonemic grouping is applied only to the UBM corpus (Table 10), the pairs accuracy-AUC are generally lower than in the rest of the trials. Best values using GITA as the test corpus are 74%–0.84 for the nasal category, followed closely by plosives. When Neurovoz is the testing corpus, the maximum values are 81%–0.83 with vowel grouping, although in this case fricative grouping provides better AUC (0.85). Finally, CzechPD as the testing corpus provides values of 82%–0.95 for vowel grouping. In these cases, vowel segments tend to be more decisive. However, only DDK tasks were used in the cross-corpora trials. Since DDK tasks are not phonetically balanced, no conclusions about the relevance of the different phonemic groups can be obtained. Nevertheless, the results of these trials suggest that the proposed approaches can generalize and are not restricted to a single corpus.

The differences between patients and controls are probably related to a smaller VSA in patients caused by an incomplete articulation of the vowels, as explained in the introduction of this paper, which in this experimental set is indirectly characterized by the PLP features. At the same time, Rasta-PLP derivatives obtained from any acoustic segments, but especially from vowels, indirectly characterize the velocity and acceleration of articulation of the speaker. Therefore, approaches employing phonemic grouping of vowels are taking advantage of certain particularities of parkinsonian speech that have proven to be successful for the detection of PD in previous studies^21–25,27^.

To summarize, results suggest that plosive segments tend to provide better accuracy and AUC in the detection of PD, followed by vowel and fricative segments. This can be explained by two phenomena described in the introduction of this study: spirantization, affecting mainly plosives and fricatives, and VSA reduction, related to vowels.

#### Analysis of approaches

In general, although the first and second approaches (*raw-phon* and *phon-phon*) help to reveal which type of phonemic category is more relevant for the detection of PD within the proposed schemes, the third one (*phon-raw*) outperforms the other two in terms of accuracy and AUC, as it can be observed in Table 6. This is the only approach in which both corpora, GITA and Neurovoz, provide better results than the baseline, since the first two approaches produce improvements only when employing GITA.

One possible reason why the third proposed approach provides better results is the fact that the phonemic grouping in the UBM corpus produces GMM-UBM classifiers that are more precise for the selected phonemic grouping but are still modeling speech from all of the acoustic segments in the parkinsonian corpora, as explained in the methodology. This means that in the third approach, unlike in the other two, no acoustic segment is discarded in the adaptation-testing corpus and all can contribute with complementary information to differentiate between the two classes.

In general, the results of this study suggest that GMM-UBM techniques, while being simpler than other state-of-the-art schemes such as Deep Neural Networks, demonstrate a good performance even with small corpora and provide generalization.

#### Speech tasks

Although the best results in this study are obtained with the DDK test, this occurs in the CzechPD corpus, in which this is the only available task. Focusing in the other two parkinsonian corpora, TDU always provide the best accuracy and AUC results. The reason for that lies in the fact that TDU contain more speech variability than DDK tasks and, at the same time, create more enclosed and precise models than monologues, as the type and number of phonemes in the training and testing utterances are always the same, allowing for a better comparison between classes.

#### Other considerations

In this study, the best results were obtained with CzechPD in both cross-validation and cross-corpora trials. The differences between CzechPD results and those for the other corpora can be explained by the fact that CzechPD only contains male speakers and the models obtained in the cross-validation trials are male-specific. Likewise, as Neurovoz contains more male than female subjects, the cross-corpora models adapted using Neurovoz plus GITA are also more male-specific and more suitable to be tested with CzechPD. Also, CzechPD only includes untreated patients, most of them in an early stage, and the lack of treatment can contribute to a better detection.

On the other hand, and in relation to the results obtained for the plosive and fricative categories, the causes of the misarticulation of plosives and fricatives can be related not only to motor disturbances but to the self-perception of the duration of occlusion lengths in phonemes. To this respect, it has been previously reported that patients are inclined to perceive occlusion lengths as longer than they really are, causing errors in the identification of phonemes^45^. Therefore, one hypothesis is that these perception impairments can aggravate misarticulation during speech production as patients are not perceiving their own articulatory errors. Another hypothesis is that the mechanisms related to speech production and perception are related to cortical areas affected by the disease^45^.

Finally, each labelled acoustic segment from the TDU in this study has been considered to canonically belong to a certain phonemic category (i. e. category with the same manner of articulation) for all the speakers. This is correct for some groups of phonemes such as vowels or nasals, but it can be controversial within other categories such as plosives, since two speakers without speaking impairments can pronounce the same phoneme differently in the same sentence context due to cultural or regional varieties of the language. Although in the present work we assumed that most of the speakers had a similar articulatory behavior, the results for some manner classes (especially plosives and fricatives) could be different in different populations.

#### Future work

In future work, new corpora in other languages need to be tested in order to evaluate the language dependence of the proposed methods, considering that different languages will entail the use of different FAM. In general, this methodology can be applied to other languages so as to design language-specific protocols or diagnosis systems that focus on the most relevant phonetic groups. In the same sense, new trials based on male- and female-specific models must be addressed.

Regarding the frame selection techniques, new types of phonemic groupings must be proposed, focused on the transitions of phonemes or on representative articulatory points such as stop release or vowel transitions among others.

Also, the use of telephonic speech to train the UBM in the proposed approaches must be assessed in the future. The motivation is that there are corpora containing telephonic speech in Spanish such as FisherSP that have more hours of recordings than Albayzin, and it is unclear to which extent a larger amount of data could help providing better GMM-UBM at the expense of band-width limitation to 300–3400 Hz, noise and distortion in the corpus employed to create the UBM.

Additionally, the distinction between PD and other neurological conditions such as Huntington Disease or Friedreich’s Ataxia by means of speech analysis systems remains to be investigated, as this might lead to a significant reduction in the diagnosis uncertainty and time.

## Conclusions

This work presents three different approaches to detecting PD from speech, based on the joint use of GMM-UBM schemes and phonemic categorization. The methodological framework proposed in this paper goes deeper into the relevance of the different manner classes in the detection of PD.

The approach *phon-raw* based on phonemic grouping exclusively in the UBM corpus and employing TDU as input material provides the best results. This technique is revealed as a new scheme to focus attention on certain classes of phonemic segments of the speech during the creation of GMM-UBM models, but without discarding the rest of the speech signal; this approach provides better results than the other techniques studied in this work. Considering this approach, cross-validation trials (k-folds) provide accuracies between 85% and 94%, with AUC between 0.91 and 0.98, while cross-corpora trials provide accuracies between 75% and 82% with AUC between 0.84 and 0.95, depending on the corpora employed to adapt and test the final models. Likewise, this method produces a relative improvement of accuracy up to 7.6% in the cross-validation trials and 8.0% in the cross-corpora trials (with respect to the baseline), depending on the corpora used to adapt and test the models. In the same sense, employment of TDU produces more consistent and accurate models than the use of monologues or DDK tasks.

The use of cross corpora trials in this study is new; these types of trials are almost non-existent in the studies using speech technologies to detect PD. At the same time, results from these trials suggest that the proposed methodologies can generalize and are not highly dependent on the corpus used to adapt the UBM models.

Also, results suggest that the proposed methods can be clinically useful for patients suffering from PD in the early stages, since even in the CzechPD corpus, where most of the patients are newly diagnosed, accuracy in the cross-validation and cross-corpora trials reaches 94% and 82%, respectively.

Finally, results suggest that plosive, vowel and fricative segments (in this order) are the most relevant for PD detection employing the proposed schemes. These findings are related to phenomena reported in previous work, such as spirantization or VSA reduction in parkinsonian patients.

## Data Availability

In order to facilitate the reproducibility of this work and its comparison with further studies, the characterization of all the text-dependent utterances from Neurovoz employed in this work (Rasta-PLP coefficients) and associated metadata are included in the following repository: 10.5281/zenodo.3401685.
